# Estimativa de Produtividade Perdida Atribuída a Doenças Cardiovasculares na América do Sul

**DOI:** 10.36660/abc.20230521

**Published:** 2024-03-20

**Authors:** Tayna Felicissímo Gomes de Souza Bandeira, Gabriela Bittencourt Gonzalez Mosegui, Cid Manso de Mello Vianna, Alfonso Jesús Gil López

**Affiliations:** 1 Universidade Federal Fluminense – PPG-GAFAR Rio de Janeiro RJ Brasil Universidade Federal Fluminense – PPG-GAFAR, Rio de Janeiro, RJ – Brasil; 2 Universidade do Estado do Rio de Janeiro Política, Planejamento e Administração em Saúde Rio de Janeiro RJ Brasil Universidade do Estado do Rio de Janeiro – Política, Planejamento e Administração em Saúde, Rio de Janeiro, RJ – Brasil; 3 Universidad de la Rioja – Departamento de Economia e Empresa Departamento de Economia e Empresa Logrono Espanha Universidad de la Rioja – Departamento de Economia e Empresa, Logrono, La Rioja – Espanha

**Keywords:** Efeitos Psicossociais da Doença, Doenças Cardiovasculares, Anos de Vida Ajustados pela Incapacidade, América do Sul

## Abstract

**Fundamento:**

As doenças cardiovasculares (DCV) têm ônus sanitário e econômico significativos. Na América do Sul (AS), a perda de produtividade relacionada a estas enfermidades ainda não foi bem explorada.

**Objetivo:**

Estimar os anos de vida produtiva perdidos (AVPP) e a perda de produtividade relacionados a mortalidade prematura associada as DCV na AS, em 2019.

**Métodos:**

Empregou-se dados de mortalidade disponíveis no Global Burden of Disease Study 2019 na estimativa da carga de doença atribuível a DCV. Para os cálculos monetários da perda da produtividade usou-se uma *proxy* da abordagem de capital humano. Estratificou-se por sexo, nas faixas etárias de trabalho.

**Resultados:**

O número total de mortes por DCV na AS no ano de 2019 foi de 754.324 e os AVPP foram 2.040.973. A perda permanente de produtividade total foi de aproximadamente US$ 3,7 bilhões e US$ 7,8 bilhões em paridade do poder de compra, equivalente a 0,11% do produto interno bruto. O custo por morte foi de US$ 22.904, e a razão desse custo por óbito, entre homens e mulheres foi 1,45. A variação dos cenários aponta robustez nas estimativas, mesmo com diferenças importantes entre os países.

**Conclusões:**

As DCV impõem um ônus econômico significativo a este bloco de países. A caracterização deste fardo pode amparar os governos na alocação de recursos destinados ao planejamento e execução de políticas e intervenções sanitárias, sejam de promoção, prevenção ou recuperação.

**Figure:**
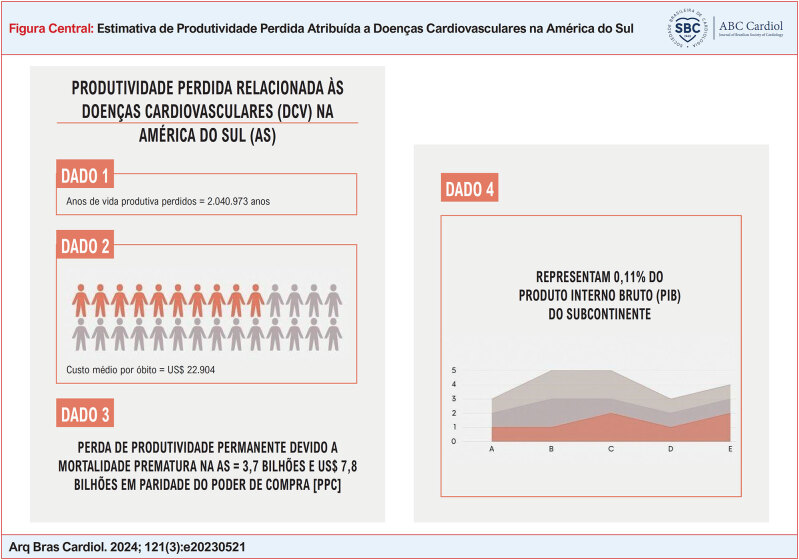


## Introdução

As doenças cardiovasculares (DCV) são uma das principais causas de morbimortalidade em todo o mundo, responsáveis por 17,9 milhões de óbitos em 2019.^1^ A carga destas doenças na América Latina e Caribe, especialmente na América do Sul (AS), cresceu ao longo dos anos, amparada por mudanças epidemiológicas, demográficas e de estilo de vida.^1^ Cerca de 75% destes óbitos ocorre em pacientes em idade economicamente ativa e países de baixa e média renda. A AS é particularmente afetada, com um ônus econômico direto e indireto para indivíduos e sociedade.^2 - 4^

A perda de produtividade em função dessas enfermidades ainda possui lacunas na América Latina e Caribe.^5 , 6^ Os custos indiretos incluem as perdas na produtividade temporária ou permanente no trabalho. Os custos da mortalidade estão associados aos óbitos prematuros consequentes da doença. Segundo Siqueira et al.,^3^ as mortes prematuras por DCV no Brasil, em 2015, custaram US$ 6.535.069.771, representando 61% do custo total estimado para as DCV. Elas são a principal causa de mortalidade e morbidade na Argentina, representando 34,2% das mortes e 12,6% dos anos potenciais de vida perdidos.^7^ Gheorghe et al.^8^ indicam a necessidade de pesquisas econômicas de qualidade para preencher as lacunas existentes. Uma análise destas perdas para a AS, possibilitaria um entendimento adicional ao reconhecimento de prioridades em saúde e na tomada de decisões sobre a prevenção, diagnóstico e no tratamento destas doenças. Este estudo estimou esta produtividade perdida considerando uma perspectiva social sobre a carga das DCV na AS, em 2019.

## Métodos

### Tipo de estudo

Trata-se de um estudo exploratório, de base populacional, transversal, que estimou as perdas permanentes de produtividade relacionadas às DCV na população em idade laboral, nos países da AS, em 2019.

### Fontes de informação

As métricas utilizadas neste estudo foram obtidas do Instituto de Métricas e Avaliação em Saúde (Institute for Health Metrics and Evaluation), por meio do Global Health Data Exchange (GHDx),^9^ um catálogo de censos e estatísticas vitais relacionadas à saúde. Essa ferramenta sintetiza diversas fontes de dados utilizadas na estimativa de mortalidade, causas de morte, doenças e fatores de risco do Estudo da Carga Global de Doenças, Lesões e Fatores de Risco (Global Burden of Disease – GBD) de 2019.^9^ Nela, modelos estatísticos são empregados na obtenção de melhores estimativas, permitindo comparação entre países, regiões e dados subnacionais através de padronização na qualidade dos dados de mortalidade dos locais. Possibilita ainda análise de tendências populacionais, pois os dados das séries temporais são ajustados e padronizados, permitindo comparabilidade ao longo do tempo. Foram levantadas mortes para cada país (localização), causa, faixa etária e sexo, em números absolutos e taxas por 100.000 habitantes.^9^

Obteve-se os dados econômicos como a participação da força de trabalho (FT), taxa de emprego, taxa de desemprego e o salário-mínimo mensal de cada um dos países, em dólares e em paridade do poder de compra (PPC), no site da International Labour Organization,^10^ assim como a população em idade laboral, por sexo e faixa etária. As faixas etárias usadas foram 15-24 anos e 25 anos ou mais. As idades de aposentadoria foram obtidas a partir de bases de dados como a Associação Internacional de Seguridade Social, (ISSA)^11^ a Comissão Econômica para a América Latina e o Caribe (CEPAL),^12^ o Banco Interamericano de Desenvolvimento (BID), a Organização para Cooperação e Desenvolvimento Econômico (OCDE) e o Banco Mundial (BM).^13^

### Estimativa da perda de produtividade

Utilizou-se uma *proxy* da abordagem do capital humano (ACH)^2 , 14 , 15^ para estimar a perda de produtividade permanente associada às DCV na AS, nas faixas etárias economicamente ativas (15 a 69 anos), por sexo, para o ano de 2019. Esse cálculo é o produto da multiplicação do tempo perdido pelo salário de mercado.^2 , 14 , 16^ Para cada morte por DCV em pessoas em idade laboral, os anos de vida produtiva perdidos (AVPP) foram calculados como a diferença entre a idade de aposentadoria e a idade de morte por DCV (com base no ponto médio da faixa etária).^17^ Calculou-se para todas as faixas etárias (até a idade limite da aposentadoria), e o valor encontrado foi multiplicado pelo número de pessoas que morrem dentro dessa faixa etária. Este produto foi agregado em intervalos de 15 a 24 e maiores de 25 anos. Extraiu-se dos bancos de dados do BM^18^ e do ILOSTAT^10^ o número de pessoas na FT e fora da força de trabalho (FFT), por sexo e faixa etária, em cada país. Pessoas FFT estão em idade ativa, mas que durante o período especificado, encontravam-se desempregadas ou não estavam empregadas (informalidade). Indivíduos com 15 anos ou mais são considerados em idade economicamente ativa.^10 , 18^ O limite máximo foi dado pela idade da aposentadoria. Somou-se os valores da FT e FFT criando-se um denominador para a proporção da FT, cujo numerador era a própria FT. A taxa de emprego foi calculada a partir da taxa de desemprego.^15^

O custo total da perda permanente de produtividade relacionada as DCV na AS foi calculada como o produto entre: somatório dos AVPP para cada óbito, a proporção da FT, a taxa de emprego e o salário-mínimo anual, expresso em dólares e em PPC de cada país, nas faixas economicamente ativas.^19^ Cálculos por sexo também foram efetuados. O uso dos valores em PPC permitiu comparações de renda mais robustas. Foi incorporada uma taxa de crescimento para os salários-mínimos de 2% ao ano.^2 , 15^ Uma taxa de desconto de 3% foi aplicada.^2 , 20^ O desconto estima aquilo que um custo ou o resultado realizado em um momento t_1_ representa em relação ao mesmo resultado ou custo ocorrido no momento presente t_0_.^20^

Tal qual Mosegui et al.,^21^ desenhou-se cenários alternativos para análise das perdas de produtividade nestes países. Adicionou-se 5 anos à idade de aposentadoria, alterou-se a tendência dos óbitos empregando os valores dos intervalos de confiança (IC) inferior e superior produzidos no GHDx^9^ e modificou-se as taxas de desconto em 0% e 6%.

### Indicadores

Os resultados foram apresentados por meio dos indicadores: (a) custo total de perda de produtividade; (b) custo da perda de produtividade por morte (custo total dividido pelo número de mortes por DCV em pessoas em idade ativa); (c) razão entre os custos masculinos e femininos, por óbito (custo masculino por morte dividido pelo custo feminino por morte); e (d) custo total da perda de produtividade como proporção do produto interno bruto (PIB) (custo total dividido pelo PIB de 2019 específico do país).^2 , 15 , 22^ Os resultados foram convertidos para dólares americanos (US$)^23^ de 2019 aplicando-se taxas de câmbio de PPC.^19^ Usou-se o *Microsoft Excel* ® versão 365 para as análises e cálculos.

### Questões éticas

Este estudo utilizou exclusivamente dados secundários provenientes de fontes de domínio público, sem qualquer identificação nominal, e respeitou os princípios éticos estabelecidos na Resolução do Conselho Nacional de Saúde nº 466, de 12 de dezembro de 2012, dispensando análise do Comitê de Ética em Pesquisa.^24^

## Resultados

A AS é considerada um subcontinente do continente americano cuja extensão é de 17.819.100 km², com 6% da população mundial e um PIB de US$3.414.784.417. São 12 países com diversidade de idiomas, população, expectativa de vida, indicadores econômicos e sociodemográficos.^22^ Em 2019 foram notificados 754.324 óbitos (4,06% das mortes globais), com diferenças importantes entre sexo e faixas etárias. Na Tabela 1 encontram-se os dados socioeconômicos, demográficos e os óbitos por DCV nos países do bloco.Os AVPP, a proporção da FT, a taxa de emprego e as produtividades perdidas nominal e em PPC (US$), para ambos os sexos, encontram-se na Tabela 2 . Os cálculos de produtividade perdida relacionada às DCV na AS não consideraram a Venezuela devido à ausência de dados. Na AS foram notificados 171.757 óbitos nas faixas etárias produtivas, 22,77% do total de mortes relacionadas às DCV. Quanto aos AVPP, perdeu-se 2.040.973 anos, afetando mais os homens (1.289.759 anos) do que as mulheres (751.214 anos). A perda de produtividade nominal foi de aproximadamente US$ 3,7 bilhões e cerca de US$ 7,8 bilhões em PPC.

**Tabela 1:** Linha de base demográfica, mortalidade por DCV e dados econômicos para a América do Sul, 2019

País	População total a	Mortes por DCV * (n) b	Expectativa de vida ao nascer (anos) a	Salário-mínimo mensal em US$ (2019) c	Salário-mínimo em PPC US$ (2019) c	PIB (milhões US$) d	Sexo	Pessoas FFT por 1.000 c	Pessoas na FT por 1.000 c	TD (%) c	Idade aposentadoria (2019) d , e , f
Argentina	44.938.712	101.724	76,6	350	813	452.819	F	8.737	8.796	10,7	65
							M	4.671	11.562	9,2	65
Bolívia	11.513.102	15.904	71,5	307	786	40.895	F	1.573	2.435	4,4	60
							M	768	3.210	3,4	60
Brasil	211.049.519	397.993	75,8	253	443	1.873.288	F	39.871	4.5756	14,5	65
							M	22.461	58.621	10,1	65
Chile	18.952.035	30.1145	80,1	428	726	278.585	F	3.937	3.858	8	65
							M	2.229	5.233	6,7	65
Colômbia	50.339.443	72.629	77,2	252	602	323.110	F	8.827	11.235	12,8	54
							M	3.840	15.050	7,9	59
Equador	17.373.657	21.464	77,0	394	755	108.108	F	2.887	3.443	4,6	65
							M	1.361	4.870	3,3	65
Guiana	782.775	2.430	69,9	212	437	5.174	F	163	120	15,1	60
							M	96	186	12,5	60
Paraguai	7.044.639	9.912	74,2	351	874	37.925	F	982	1.477	8,3	65
							M	396	2.134	5,4	65
Peru	32.510.462	29.215	76,7	279	534	228.326	F	3.678	8.600	3,7	65
							M	1.823	10.198	3,1	65
Suriname	581.363	1.423	71,6	234	634	4.221	F	117	97	11,1	60
							M	70	141	5,7	60
Uruguai	3.461.731	10.003	77,9	444	616	61.231	F	636	807	10,5	70
							M	373	937	7,2	70
Venezuela, RB	28.515.829	61.510	72,0	DI	DI	DI	F	6.794	3.850	DI	55
							M	2.986	7.071	DI	60
AS	427.063.267	754.324	75,1			3.413.682					

Fontes:

a BM^22^

b GHDx^9^

c OCDE^25^

d BID

e OCDE e

f CEPAL.^13^

DCV: doenças cardiovasculares; DI: dados indisponíveis; F: feminino; FFT: fora da força de trabalho; FT: força de trabalho; M: masculino; PIB: produto interno bruto; PPC: paridade do poder de compra; TD: taxa de desemprego.

* Todas as faixas etárias, exceto de 0 a 1 ano.

**Tabela 2:** Estimativa da produtividade perdida devido a DCV, por sexo e faixa etária, na América do Sul, 2019

Países		Avpp (anos)		Proporção da ft		Te		Produtividade perdida em salário-mínimo ppc 2019 (us$)			Produtividade perdida em salário-mínimo nominal 2019 (us$)		
		M	H	M	H	M	H	M	H	Total	M	H	Total
**Argentina**								**296.791.198**	**917.730.803**	**1.214.522.001**	**115.026.626**	**355.034.032**	**470.060.658**
	**15 a 24**	5.291	7.787	0,32	0,46	0,71	0,76	11.728.656	26.560.125	38.288.781	4.103.507	7.631.682	11.735.189
	**25+**	57.745	125.925	0,55	0,78	0,92	0,93	285.062.542	891.170.678	1.176.233.220	110.923.119	347.402.350	458.325.469
**Bolívia**								**91.079.535**	**131.432.052**	**222.511.587**	**32.599.388**	**46.994.493**	**79.593.881**
	**15 a 24**	2.387	3.210	0,42	0,57	0,9	0,92	8.508.757	15.876.815	24.385.572	2.760.745	5.150.824	7.911.569
	**25+**	13.272	13.891	0,68	0,9	0,97	0,98	82.570.778	115.555.237	198.126.015	29.838.643	41.843.669	71.682.312
**Brasil**								**1.258.750.573**	**2.991.931.297**	**4.250.681.870**	**660.929.012**	**1.572.432.934**	**2.233.361.946**
	**15 a 24**	27.206	44.470	0,50	0,62	0,68	0,77	49.173.646	112.859.096	162.032.742	22.837.746	52.414.123	75.251.869
	**25+**	473.440	776.468	0,54	0,75	0,89	0,93	1.209.576.927	2.879.072.201	4.088.649.128	638.091.266	1.520.018.811	2.158.110.077
**Chile**								**74.349.711**	**239.794.767**	**314.144.478**	**40.661.875**	**131.050.476**	**171.712.351**
	**15 a 24**	886	1.695	0,29	0,34	0,8	0,81	1.791.295	4.067.723	5.859.018	858.679	1.950.514	2.809.193
	**25+**	16.584	36.904	0,54	0,78	0,93	0,94	72.558.416	235.727.044	308.285.460	39.803.196	129.099.962	168.903.158
**Colômbia**								**127.429.514**	**353.558.605**	**480.988.119**	**29.810.206**	**111.061.199**	**140.871.405**
	**15 a 24**	4.363	6.258	0,43	0,58	0,75	0,84	10.165.588	22.024.497	32.190.085	3.091.135	7.512.333	10.603.468
	**25+**	30.570	56.771	0,59	0,86	0,9	0,94	117.263.926	331.534.108	448.798.034	26.719.071	103.548.866	130.267.937
**Equador**								136.563.971	347.182.550	483.746.521	64.739.035	**163.477.167**	**228.216.202**
	**15 a 24**	3.584	7.480	0,34	0,53	0,88	0,93	9.715.173	33.402.974	43.118.147	4.116.405	14.181.808	18.298.213
	**25+**	23.662	40.621	0,61	0,87	0,97	0,98	126.848.798	313.779.576	440.628.374	60.622.630	149.295.359	209.917.989
**Guiana**								**5.445.540**	**13.658.976**	**19.104.516**	**3.533.192**	**9.052.876**	**12.586.068**
	**15 a 24**	300	329	0,4	0,57	0,67	0,77	422.095	756.740	1.178.835	187.792	337.062	524.854
	**25+**	2.448	3.862	0,43	0,7	0,91	0,91	5.023.445	12.902.236	17.925.681	3.345.400	8.715.814	12.061.214
**Paraguai**								**77.488.198**	**180.306.355**	**257.794.553**	**28.595.124**	**66.565.135**	**95.160.259**
	**15 a 24**	710	1.272	0,58	0,66	0,8	0,88	3.450.738	7.750.553	11.201.291	1.126.878	2.531.491	3.658.369
	**25+**	10.179	18.846	0,73	0,9	0,95	0,97	74.037.460	172.555.802	246.593.262	27.468.246	64.033.644	91.501.890
**Peru**								129.370.320	302.746.706	**432.117.026**	**60.950.039**	**141.647.988**	**202.598.027**
	**15 a 24**	5.073	8.912	0,47	0,71	0,92	0,93	14.056.892	37.707.503	51.764.395	5.965.248	16.021.045	21.986.293
	**25+**	28.541	47.421	0,65	0,89	0,97	0,98	115.313.428	265.039.203	380.352.631	54.984.791	125.626.943	180.611.734
**Suriname**								**3.906.368**	**10.571.886**	**14.478.254**	**1.986.624**	**5.481.103**	**7.467.727**
	**15 a 24**	106	118	0,28	0,47	0,6	0,81	135.368	342.785	478.153	45.550	115.601	161.151
	**25+**	1.045	1.899	0,51	0,73	0,93	0,97	3.771.000	10.229.101	14.000.101	1.941.074	5.365.502	7.306.576
**Uruguai**								**27.863.176**	**77.418.106**	**105.281.282**	**18.535.738**	**51.661.907**	**70.197.645**
	**15 a 24**	289	403	0,42	0,52	0,68	0,76	610.652	1.176.064	1.786.716	350.061	673.950	1.024.011
	**25+**	6.719	14.137	0,59	0,76	0,93	0,96	27.252.524	76.242.042	103.494.566	18.185.677	50.987.957	69.173.634
**Venezuela**													
	**15 a 24**	4.692	3.474	0,19	0,51	0,83	0,88	DI	DI	DI	DI	DI	DI
	**25+**	32.122	67.606	0,41	0,76	0,93	0,93	DI	DI	DI	DI	DI	DI
**América do Sul**		**751.214**	**1.289.759**					2.229.038.104	5.566.332.103	7.795.370.207	1.057.366.859	2.654.459.310	**3.711.826.169**

Fonte: Autoria própria.

AVPP: anos de vida produtiva perdidos; DI: dados indisponíveis; FT: força de trabalho; H: homens; M: mulheres; PPC: paridade do poder de compra; TE: taxa de emprego.

Brasil e Argentina apresentaram os maiores valores de AVPP, aproximadamente 1,3 milhão e 196.748 anos respectivamente, enquanto os menores registros foram do Suriname (3.168 anos) e Guiana (6.939 anos). Os custos de perda de produtividade total em PPC equivaleram a US$ 4.250.681.870 no Brasil, 54,5% do total da AS, e US$ 1.214.522.001 na Argentina. Suriname e Guiana tiveram perdas de produtividade em PPC mais baixas, US$14.478.254 e US$19.104.516. As perdas nominais em salários-mínimos foram de US$ 2.233.361.946 no Brasil, representando 60,2% destas perdas para o subcontinente, e US$470.060.658 na Argentina. Suriname apresentou a menor perda nominal, US$ 7.467.727, seguido pela Guiana, US$12.586.068. Dentro das faixas etárias e por sexo, em todos os países, as estimativas de custo foram maiores para homens acima de 25 anos.

Na Tabela 3 encontram-se o custo da perda de produtividade por óbito, razão dos custos por morte de homens e mulheres e o custo total da produtividade perdida como percentual do PIB.

**Tabela 3:** Estimativa do custo por morte (US$, 2019), razão dos custos por morte (razão H/M) e custo total como % do PIB, por DCV, na América do Sul, 2019

	Custo por morte (US$, 2019)			Razão dos custos por morte e sexo (H/M)	Custo total como % do PIB
	M	H	Total		
**Argentina**	20.884	28.899	26.418	1,38	0,10
**Bolívia**	26.106	33.574	30.053	1,29	0,19
**Brasil**	15.612	22.507	19.905	1,44	0,12
**Chile**	24.129	36.271	32.409	1,50	0,06
**Colômbia**	13.259	21.838	19.208	1,65	0,04
**Equador**	32.745	49.850	43.417	1,52	0,21
**Guiana**	14.120	22.337	19.200	1,58	0,24
**Paraguai**	31.127	38.729	36.081	1,24	0,25
**Peru**	26.396	39.169	34.192	1,48	0,09
**Suriname**	17.978	26.410	23.480	1,47	0,18
**Uruguai**	28.459	38.369	35.138	1,35	0,11
**Venezuela**	DI	DI	DI	DI	DI
**América do Sul**	**17.848**	**25.816**	**22.904**	**1,45**	**0,11**

Fonte: Autoria própria.

DI: dados indisponíveis; H: homens; M: mulheres.

A perda de produtividade permanente nos 11 países analisados representou 0,11% do PIB combinado, em 2019; com custos variando de 0,04% e 0,06%, na Colômbia e Chile, a 0,25% e 0,24% no Paraguai e Guiana, respectivamente. Os custos por óbito na Colômbia (US$ 19.208), Guiana (US$ 19.200) e Brasil (US$ 19.905) foram quase duas vezes menores do que no Paraguai (US$ 36.081) e Uruguai (US$ 35.138). Para a AS, o custo por morte foi de US$ 22.904.

As estimativas feitas por sexo apontam mais mortes entre homens do que mulheres em idade laboral, por DCV, na AS, com variações importantes na razão H:M, como 1,24 no Paraguai e 1,65 na Colômbia. Os custos das perdas de produtividade em PPC e nominais também mostraram diferenças significativas. No Chile e na Argentina, os valores em PPC para homens foram 3,2 (US$ 239.794.767) e 3,1 (US$ 917.730.803) vezes maiores do que os das mulheres, respectivamente. A Bolívia apresentou menor diferença dos custos em PPC entre os sexos (homens US$ 131.432.052 e mulheres US$ 91.079.535). As perdas nominais em salários-mínimos foram 3,7 e 3,2 vezes maiores no sexo masculino, na Colômbia e Chile (US$ 111.061.199 e US$ 131.050.476) comparadas ao feminino (US$ 29.810.206 e US$ 40.661.875). Já a Bolívia e o Peru tiveram menores diferenças entre homens e mulheres: 1,44 e 2,32 vezes os valores estimados, respectivamente.

Cenários alternativos foram construídos ( Tabela 4 ) a partir dos resultados da Tabela 2 , variando-se: (a) valores da taxa de desconto, (b) idade de aposentadoria e (c) número de óbitos, este último usando os IC superior e inferior presentes no GHDx.^9^

**Tabela 4:** Perdas de produtividade percentuais relacionadas ao cenário base com variação das taxas de desconto, idade de aposentadoria e número de mortes

Países	Sem desconto	Desconto 6%	Aposentadoria real após 5 anos da legal	Número absoluto de óbitos (IC superior)	Número absoluto de óbitos (IC inferior)
	(%)	(%)	(%)	(%)	(%)
**Argentina**	33	-16	48	15	-10
**Bolívia**	34	-20	41	59	-47
**Brasil**	31	-19	41	6	-6
**Chile**	28	-18	46	9	-12
**Colômbia**	30	-18	50	37	-29
**Equador**	40	-22	35	37	-28
**Guiana**	34	-21	30	34	-28
**Paraguai**	32	-19	41	42	-32
**Peru**	43	-24	31	50	-37
**Suriname**	31	-20	32	32	-27
**Uruguai**	29	-18	45	13	-12

Fonte: Autoria própria.

IC: intervalo de confiança.

Estimou-se as perdas laborais ocasionadas por óbitos precoces usando-se diferentes taxas de desconto. As perdas de produtividade diminuíram para todos os países com desconto de 6%. Quanto à mudança na idade de aposentadoria, as variações foram positivas, apontando para um crescimento dos custos relacionados a produtividade perdida permanente, pois houve aumento do tempo de contribuição na FT. Alterou-se a mortalidade usando-se os IC inferior e superior do GHDx. Ao aplicarmos o IC superior do número de mortes, a estimativa da produtividade perdida aumenta e com o inferior, ela diminui para toda a AS.

## Discussão

Os resultados desta análise apontam para um custo total da perda de produtividade em razão das DCV, nas economias da AS, de cerca de US$ 3,7 bilhões (US$ 7,8 bilhões em PPC), representando 0,11% do PIB combinado desses países (oscilando entre 0,04% e 0,24%). Os custos por morte alcançaram US$ 22.904 em 2019. Com mercados muito distintos quanto às horas trabalhadas e aos salários, a comparação entre o PIB e as perdas de produtividade evidenciam o potencial impacto na dimensão econômica e as perdas sociais produzidas a partir de suas mortes prematuras. Regiões de menores índices socioeconômicos têm maiores taxas de mortalidade.^6 , 8^

Os países analisados possuem AVPP e estimativas de produtividade perdida distintas, o que pode ser explicado pelas diferenças populacionais e econômicas (PIB, FT, desemprego e idade de aposentadoria).^10 , 13 , 22^ A ACH vem sendo utilizada no cálculo e na interpretação dos custos das mortes prematuras em diferentes regiões e grupos de enfermidades.^2 , 14 , 21^ A alteração dos cenários sugere que elementos locais como a idade de aposentadoria, mudanças na FT e óbitos associados as DCV tem relevância na análise dos resultados.^2 , 21^

Nossa proposta diferiu da de Azambuja et al.^26^ que estimaram os custos diretos e indiretos referentes aos casos de DCV grave no Brasil, para 2004. Os autores apontaram um custo anual de R$ 30,8 bilhões, onde 55,2% resultariam da perda de produtividade em pacientes acima de 35 anos (R$ 17.013.350.772,00). Apontaram custos diretos correspondentes a 8% do gasto total do país com saúde e 0,52% do PIB (US$ 602 bilhões). Considerando o valor médio do dólar para 2004 (R$ 3,05),^23^ a estimativa US$ 5.578.147.794 dos custos indiretos para pacientes graves acima de 35 anos é menor do que a encontrada em nosso estudo (US$ 2.233.361.946), onde incorporou-se uma população ativa mais jovem e casos não graves. Os gastos como percentual do PIB (0,12%) refletem as diferenças metodológicas, mas sugerem uma proporcionalidade entre os resultados destes estudos. As bases de dados, metodologias, usos de outros custos e taxas de desconto podem ser responsáveis pela diferença nestes resultados.

Brasil e Argentina foram os países mais afetados quanto às perdas de produtividade, destacando o impacto negativo das DCV no desenvolvimento econômico. A expressão dos produtos em PPC lhes confere maior homogeneidade e comparabilidade ( Tabela 2 ). Foram poucos estudos que abordaram a produtividade perdida relacionada às DCV na AS ou mesmo na América Latina e Caribe.^3 , 4 , 7 , 26^ Não foram encontrados trabalhos expressando resultados de produtividade perdida das DCV com PPC.

Mosegui et al.^27^ calcularam as perdas de produtividade permanentes relacionadas ao câncer na AS usando a mesma abordagem (ACH) e base de dados (GBD 2019). Os autores relataram 192.240 óbitos e 2.463.155 AVPP. A perda total de produtividade permanente foi de US$ 4,4 bilhões e US$ 9,4 bilhões PPC – 0,13% do PIB da região. O custo total por morte foi de US$ 23.617. As DCV matam quase quatro vezes mais pessoas do que as neoplasias.^6 , 28^ Os custos por óbito não diferiram muito dos encontrados em nosso estudo (US$ 22.904), enquanto a razão deste custo por morte, entre sexos foi menor (1,28) do que os 1,45 estimados para as DCV. Nossas estimativas de custos/morte parecem mais baixas do que as relatadas em outros locais e regiões.^3 , 4 , 7 , 26^ É necessária cautela ao compararmos alguns achados, devido às diferenças metodológicas, populacionais e econômicas existentes.

Algumas limitações deste estudo merecem ser examinadas. Os dados utilizados no cálculo dos AVPP foram provenientes do GBD 2019, presentes no GBDx,^9^ alternativa oportuna frente à escassez de bases e estudos com qualidade metodológica adequada na região. O uso de dados sanitários e econômicos globais^9 , 10 , 12 , 18^ como alternativa aos dados nacionais, geralmente mais consistentes, relaciona-se à dificuldade de encontrá-los, como observado para a Venezuela. Segundo Hofmarcher et al.,^29^ análises de custo multinacionais são difíceis de realizar, principalmente devido a barreiras associadas à identificação de informações.

Esta análise ateve-se à perda permanente de produtividade, permitindo uma caracterização quantitativa da carga das DCV. Não se calculou as perdas temporárias ou custos diretos de saúde.^3 , 26^ Empregou-se salários-mínimos ao invés de rendimentos médios. Mosegui et al.^21^ sinalizaram que para o bloco de países da AS, salários médios e mínimos não expressam a forma como os rendimentos e salários se organizam nos distintos grupos, frente à recorrente informalidade no trabalho^10 , 12 , 18^ e às diferenças entre a idade legal de aposentadoria e a real. Apesar da ACH ser o método de cálculo de perda de produtividade mais usado,^2^ seus críticos apontam possíveis vieses nos padrões de rendimentos, onde grupos desfavorecidos (jovens, mulheres), com ganhos menores, têm uma desvalorização de sua produtividade perdida.

Pela primeira vez são estimados ônus econômico relacionado às DCV em todos os países da AS, numa perspectiva adicional às análises de carga da doença. A perda de produtividade permanente associada às DCV evidenciada no estudo é expressiva e seu impacto individual e social sugere uma perda *per capita* média de US$ 22.904 na população economicamente ativa, assim como uma perda social para a AS de aproximadamente US$ 3,7 bilhões e US$ 7,8 bilhões em PPC.

## Conclusão

Fatores socioeconômicos influenciam a saúde cardiovascular, assim como as desigualdades e ineficiências nos sistemas sanitários. As DCV impõem um importante encargo econômico e sanitário aos países da AS, levando a perdas sociais e de produtividade. Nossos achados podem ser úteis na formulação e implementação de políticas públicas e estratégias efetivas de prevenção, tratamento e gestão das DCV nos países analisados.
