# Supplementary material for: Estimativa de Produtividade Perdida Atribuída a Doenças Cardiovasculares na América do Sul
Source: Arq Bras Cardiol. 2024 Mar 20;121(3):e20230521. [Article in Portuguese] doi: 10.36660/abc.20230521 (PMC12094524; doi:10.36660/abc.20230521)
Supplement: Translation

# Translation: Estimated Loss of Productivity Attributed to Cardiovascular Diseases in South America

**DOI:** 10.36660/abc.20230521i

**Authors:** Tayna Felicissímo Gomes de Souza Bandeira, Gabriela Bittencourt Gonzalez Mosegui, Cid Manso de Mello Vianna, Alfonso Jesús Gil López

**Affiliations:** 1 Rio de Janeiro RJ Brazil Universidade Federal Fluminense – PPG-GAFAR, Rio de Janeiro, RJ – Brazil; 2 Rio de Janeiro RJ Brazil Universidade do Estado do Rio de Janeiro – Política, Planejamento e Administração em Saúde, Rio de Janeiro, RJ – Brazil; 3 Logrono Espanha Universidad de la Rioja – Departamento de Economia e Empresa, Logrono, La Rioja – Espanha

**Keywords:** Cost of Illness, Cardiovascular Diseases, Disability-Adjusted Life Years, South America

## Abstract

**Background:**

Cardiovascular diseases (CVD) have significant health and economic burdens. In South America, the loss of productivity related to these diseases has not yet been well explored.

**Objective:**

Estimate the potentially productive years of life lost (PPYLL) and loss of productivity related to premature mortality associated with CVD in South America, in 2019.

**Methods:**

Mortality data available from the 2019 Global Burden of Disease Study were used to estimate the burden of disease attributable to CVD. For monetary calculations of productivity loss, a proxy of the human capital approach was used. Data were stratified by sex, in working age groups.

**Results:**

The total number of deaths due to CVD in South America in 2019 was 754,324, and the total number of PPYLL was 2,040,973. The total permanent loss of productivity was approximately US$ 3.7 billion and US$ 7.8 billion in purchasing power parity, equivalent to 0.11% of the gross domestic product. The cost per death was US$ 22,904, and the ratio between men and women for the cost per death was 1.45. The variation in scenarios indicates that the estimates are robust, even with important differences between countries.

**Conclusions:**

CVD impose a significant economic burden on countries in South America. The characterization of this burden can support governments in the allocation of resources for the planning and execution of health policies and interventions in promotion, prevention, and recovery.

## Introduction

Cardiovascular diseases (CVD) are one of the main causes of morbidity and mortality worldwide, responsible for 17.9 million deaths in 2019.^1^The burden of these diseases in Latin America and the Caribbean, especially in South America, has grown over the years, underpinned by epidemiological, demographic, and lifestyle changes.^1^Approximately 75% of these deaths occur in patients of economically active age and in low- and middle-income countries. South America is particularly affected, with a direct and indirect economic burden on individuals and society.^2 - 4^

There are still gaps regarding the loss of productivity due to these illnesses in Latin America and the Caribbean.^5 , 6^Indirect costs include temporary or permanent losses of productivity at work. Mortality costs are associated with premature deaths resulting from the disease. According to Siqueira et al.,^3^premature deaths from CVD in Brazil, in 2015, cost US$ 6,535,069,771, representing 61% of the total estimated cost of CVD. They are the main cause of mortality and morbidity in Argentina, representing 34.2% of deaths and 12.6% of potential years of life lost.^7^Gheorghe et al.^8^indicate the need for quality economic research to fill the existing gaps. An analysis of these losses in South America would promote further understanding for recognition of health priorities and decision-making regarding prevention, diagnosis, and treatment of these diseases. This study estimated this loss of productivity considering a social perspective on the burden of CVD in South America in 2019.

## Methods

### Study type

This exploratory, population-based, cross-sectional study estimated permanent losses of productivity related to CVD in the working-age population in South American countries in 2019.

### Information sources

The metrics used in this study were obtained from the Institute for Health Metrics and Evaluation, by means of the Global Health Data Exchange (GHDx),^9^a catalog of censuses and vital statistics related to health. This tool synthesizes several sources of data used to estimate mortality, causes of death, diseases, and risk factors from the 2019 Global Burden of Disease (GBD) Study.^9^It uses statistical models to obtain better estimates, allowing comparison between countries, regions, and subnational data through standardization in the quality of local mortality data. It also enables analysis of population trends, as time series data are adjusted and standardized, allowing comparability over time. Deaths were collected for each country (location), cause, age group, and sex, in absolute numbers and rates per 100,000 inhabitants.^9^

Economic data such as labor force (LF) participation, employment rate, unemployment rate, and the monthly minimum wage of each country were obtained, in dollars and purchasing power parity (PPP), from the website of the International Labour Organization,^10^in addition to the working-age population, by sex and age range. The age groups used were 15 to 24 years and 25 years and over. Retirement ages were obtained from databases such as the International Social Security Association (ISSA),^11^the Economic Commission for Latin America and the Caribbean (CEPAL),^12^the Inter-American Development Bank (IDB), the Organization for Economic Co-operation and Development (OECD), and the World Bank (WB).^13^

### Estimated loss of productivity

A proxy of the human capital approach (HCA)^2 , 14 , 15^was used to estimate the permanent loss of productivity associated with CVD in South America, in economically active age groups (15 to 69 years), by sex, for the year 2019. This calculation is obtained by multiplying the time lost by the market wage.^2 , 14 , 16^For each death from CVD in people of working age, the potentially productive years of life lost (PPYLL) were calculated as the difference between the retirement age and the age of death from CVD (based on the midpoint of the age group).^17^PPYLL were calculated for all age groups (up to the retirement age limit), and the value found was multiplied by the number of people who died within this age group. This product was aggregated in intervals of 15 to 24 and over 25 years. The numbers of people in the LF and out of the labor force (OLF), by sex and age group, in each country were extracted from the World Bank^18^and ILOSTAT^10^databases. People outside the labor force are of working age, but, during the specified period, they were unemployed or not employed (informality). Individuals aged 15 or over are considered to be of economically active age.^10 , 18^The maximum limit was established as the retirement age. The LF and OLF were added together, creating a denominator for the LF proportion, whose numerator was the LF itself. The employment rate was calculated based on the unemployment rate.^15^

The total cost of permanent loss of productivity related to CVD in South America was calculated as the product between: the sum of PPYLL for each death, the LF proportion, the employment rate, and the annual minimum wage expressed in dollars and PPP for each country, in the economically active ranges.^19^Calculations by sex were also carried out. The use of PPP values allowed for more robust income comparisons. A growth rate for minimum wage of 2% per year was incorporated.^2 , 15^A discount rate of 3% was applied.^2 , 20^The discount estimates what a cost or result at a moment t_1_represents in relation to the same result or cost occurring at the present moment t_0_.^20^

In the same manner as Mosegui et al.,^21^alternative scenarios were designed to analyze productivity losses in these countries. Five years were added to the retirement age; the trend of deaths was changed using the lower and upper confidence intervals (CI) produced in the GHDx,^9^and the discount rate was changed to 0% and 6%.

### Indicators

The results were presented using the following indicators: (a) total cost of lost productivity; (b) cost of lost productivity per death (total cost divided by the number of deaths from CVD in people of working age); (c) ratio between male and female costs, per death (male cost per death divided by female cost per death); and (d) total cost of lost productivity as a proportion of gross domestic product (GDP) (total cost divided by country-specific GDP in 2019).^2,15,22^The results were converted to US dollars (US$)^23^as of 2019, applying PPP exchange rates.^19^Microsoft Excel®, version 365 was used for analyses and calculations.

### Ethical issues

This study exclusively used secondary data from public domain sources, without any nominal identification, and it respected the ethical principles established in Brazilian National Health Council Resolution number 466, of December 12, 2012; thus, it did not require analysis by the Research Ethics Committee.^24^

## Results

South America is considered a subcontinent of the Americas. South America has an extension of 17,819,100 km², with 6% of the world's population and a GDP of US$ 3,414,784,417. It includes 12 countries with diverse languages, populations, life expectancy, and economic and sociodemographic indicators.^22^In 2019, 754,324 deaths were reported (4.06% of global deaths), with important differences between sexes and age groups. Table 1 displays socioeconomic and demographic data and deaths from CVD in South American countries. Table 2 displays the PPYLL, LF proportion, employment rate, nominal loss of productivity, and loss of productivity in PPP (US$), for both sexes. The calculations for loss of productivity related to CVD in South America did not take Venezuela into account, due to missing data. In South America, 171,757 deaths were reported in productive age groups, 22.77% of the total deaths related to CVD. As for PPYLL, 2,040,973 years were lost, affecting men (1,289,759 years) more than women (751,214 years). The nominal loss of productivity was approximately US$ 3.7 billion and approximately US$ 7.8 billion in PPP.

**Table 1:** Baseline demographic, CVD mortality, and economic data for South America, 2019

Country	Total population a	Deaths from CVD * (n) b	Life expecancy at birth (years) a	Monthy minimum wage in US$ (2019) c	Minimum wage in PPP US$ (2019) c	GDP(millions US$) d	Sex	People OLF per 1,000 c	People in LF per 1,000 c	Unemployment rate (%) c	Retirement age(2019) d , e , f
Argentina	44.938.712	101.724	76.6	350	813	452.819	F	8.737	8.796	10.7	65
							M	4.671	11.562	9.2	65
Bolivia	11.513.102	15.904	71.5	307	786	40.895	F	1.573	2.435	4.4	60
							M	768	3.210	3.4	60
Brazil	211.049.519	397.993	75.8	253	443	1.873.288	F	39.871	4.5756	14.5	65
							M	22.461	58.621	10.1	65
Chile	18.952.035	30.1145	80.1	428	726	278.585	F	3.937	3.858	8	65
							M	2.229	5.233	6.7	65
Colombia	50.339.443	72.629	77.2	252	602	323.110	F	8.827	11.235	12.8	54
							M	3.840	15.050	7.9	59
Ecuador	17.373.657	21.464	77.0	394	755	108.108	F	2.887	3.443	4.6	65
							M	1.361	4.870	3.3	65
Guyana	782.775	2.430	69.9	212	437	5.174	F	163	120	15.1	60
							M	96	186	12.5	60
Paraguay	7.044.639	9.912	74.2	351	874	37.925	F	982	1.477	8.3	65
							M	396	2.134	5.4	65
Peru	32.510.462	29.215	76.7	279	534	228.326	F	3.678	8.600	3.7	65
							M	1.823	10.198	3.1	65
Suriname	581.363	1.423	71.6	234	634	4.221	F	117	97	11.1	60
							M	70	141	5.7	60
Uruguay	3.461.731	10.003	77.9	444	616	61.231	F	636	807	10.5	70
							M	373	937	7.2	70
Venezuela	28.515.829	61.510	72.0	DI	DI	DI	F	6.794	3.850	DI	55
							M	2.986	7.071	DI	60
South America	427.063.267	754.324	75.1			3.413.682					

Sources:

a WB^22^

b GHDx^9^

c OCDE^25^

d IDB

e OCDE

f CEPAL.^13^

CVD: cardiovascular diseases; F: female; GDP: gross domestic product; LF: labor force; M: male; NA: data not available; OLF: out of labor force; PPP: purchasing power parity.

* All age groups, except from 0 to 1 year old.

**Table 2:** Estimated loss of productivity due to CVD, by sex and age group, in South America, 2019

Country		PPYLL (years)		LF proportion		Unemployment rate		Loss of productivity, considering minimum wage in PPP, 2019 (US$)			Nominal loss of productivity, considering minimum wage, 2019 (US$)		
		W	M	W	M	W	M	W	M	Total	W	M	Total
**Argentina**								**296,791,198**	**917,730,803**	**1,214,522,001**	**115,026,626**	**355,034,032**	**470,060,658**
	**15 to 24**	5,291	7,787	0.32	0.46	0.71	0.76	11,728,656	26,560,125	38,288,781	4,103,507	7,631,682	11,735,189
	**25+**	57,745	125,925	0.55	0.78	0.92	0.93	285,062,542	891,170,678	1,176,233,220	110,923,119	347,402,350	458,325,469
**Bolivia**								**91,079,535**	**131,432,052**	**222,511,587**	**32,599,388**	**46,994,493**	**79,593,881**
	**15 to 24**	2,387	3,210	0.42	0.57	0.9	0.92	8,508,757	15,876,815	24,385,572	2,760,745	5,150,824	7,911,569
	**25+**	13,272	13,891	0.68	0.9	0.97	0.98	82,570,778	115,555,237	198,126,015	29,838,643	41,843,669	71,682,312
**Brazil**								**1,258,750,573**	**2,991,931,297**	**4,250,681,870**	**660,929,012**	**1,572,432,934**	**2,233,361,946**
	**15 to 24**	27,206	44,470	0.50	0.62	0.68	0.77	49,173,646	112,859,096	162,032,742	22,837,746	52,414,123	75,251,869
	**25+**	473,440	776,468	0.54	0.75	0.89	0.93	1,209,576,927	2,879,072,201	4,088,649,128	638,091,266	1,520,018,811	2,158,110,077
**Chile**								**74,349,711**	**239,794,767**	**314,144,478**	**40,661,875**	**131,050,476**	**171,712,351**
	**15 to 24**	886	1,695	0.29	0.34	0.8	0.81	1,791,295	4,067,723	5,859,018	858,679	1,950,514	2,809,193
	**25+**	16,584	36,904	0.54	0.78	0.93	0.94	72,558,416	235,727,044	308,285,460	39,803,196	129,099,962	168,903,158
**Colombia**								**127,429,514**	**353,558,605**	**480,988,119**	**29,810,206**	**111,061,199**	**140,871,405**
	**15 to 24**	4,363	6,258	0.43	0.58	0.75	0.84	10,165,588	22,024,497	32,190,085	3,091,135	7,512,333	10,603,468
	**25+**	30,570	56,771	0.59	0.86	0.9	0.94	117,263,926	331,534,108	448,798,034	26,719,071	103,548,866	130,267,937
**Ecuador**								136,563,971	347,182,550	483,746,521	64,739,035	**163,477,167**	**228,216,202**
	**15 to 24**	3,584	7,480	0.34	0.53	0.88	0.93	9,715,173	33,402,974	43,118,147	4,116,405	14,181,808	18,298,213
	**25+**	23,662	40,621	0.61	0.87	0.97	0.98	126,848,798	313,779,576	440,628,374	60,622,630	149,295,359	209,917,989
**Guyana**								**5,445,540**	**13,658,976**	**19,104,516**	**3,533,192**	**9,052,876**	**12,586,068**
	**15 to 24**	300	329	0.4	0.57	0.67	0.77	422,095	756,740	1,178,835	187,792	337,062	524,854
	**25+**	2,448	3,862	0.43	0.7	0.91	0.91	5,023,445	12,902,236	17,925,681	3,345,400	8,715,814	12,061,214
**Paraguay**								**77,488,198**	**180,306,355**	**257,794,553**	**28,595,124**	**66,565,135**	**95,160,259**
	**15 to 24**	710	1,272	0.58	0.66	0.8	0.88	3,450,738	7,750,553	11,201,291	1,126,878	2,531,491	3,658,369
	**25+**	10,179	18,846	0.73	0.9	0.95	0.97	74,037,460	172,555,802	246,593,262	27,468,246	64,033,644	91,501,890
**Peru**								129,370,320	302,746,706	**432,117,026**	**60,950,039**	**141,647,988**	**202,598,027**
	**15 to 24**	5,073	8,912	0.47	0.71	0.92	0.93	14,056,892	37,707,503	51,764,395	5,965,248	16,021,045	21,986,293
	**25+**	28,541	47,421	0.65	0.89	0.97	0.98	115,313,428	265,039,203	380,352,631	54,984,791	125,626,943	180,611,734
**Suriname**								**3,906,368**	**10,571,886**	**14,478,254**	**1,986,624**	**5,481,103**	**7,467,727**
	**15 to 24**	106	118	0.28	0.47	0.6	0.81	135,368	342,785	478,153	45,550	115,601	161,151
	**25+**	1,045	1,899	0.51	0.73	0.93	0.97	3,771,000	10,229,101	14,000,101	1,941,074	5,365,502	7,306,576
**Uruguay**								**27,863,176**	**77,418,106**	**105,281,282**	**18,535,738**	**51,661,907**	**70,197,645**
	**15 to 24**	289	403	0.42	0.52	0.68	0.76	610,652	1,176,064	1,786,716	350,061	673,950	1,024,011
	**25+**	6,719	14,137	0.59	0.76	0.93	0.96	27,252,524	76,242,042	103,494,566	18,185,677	50,987,957	69,173,634
**Venezuela**													
	**15 to 24**	4,692	3,474	0.19	0.51	0.83	0.88	DI	DI	DI	DI	DI	DI
	**25+**	32,122	67,606	0.41	0.76	0.93	0.93	DI	DI	DI	DI	DI	DI
**South America**		**751,214**	**1,289,759**					2,229,038,104	5,566,332,103	7,795,370,207	1,057,366,859	2,654,459,310	**3,711,826,169**

Source: the authors.

CVD: cardiovascular diseases; LF: labor force; M: men; NA: data not available; PPP: purchasing power parity; PPYLL: potentially productive years of life lost; W: women.

Brazil and Argentina had the highest PPYLL values, approximately 1.3 million and 196,748 years respectively, while the lowest values were from Suriname (3,168 years) and Guyana (6,939 years). Total costs of lost productivity in PPP were equivalent to US$ 4,250,681,870 in Brazil, 54.5% of the total from South America, and US$ 1,214,522,001 in Argentina. Suriname and Guyana had lower losses of productivity in PPP, US$ 14,478,254 and US$ 19,104,516, respectively. Nominal losses in minimum wages were US$ 2,233,361,946 in Brazil, representing 60.2% of these losses for the subcontinent, and US$ 470,060,658 in Argentina. Suriname had the smallest nominal loss, US$ 7,467,727, followed by Guyana, US$ 12,586,068. Within age groups and by sex, in all countries, cost estimates were higher for men over 25 years of age.

Table 3 shows the cost of lost productivity due to death, the ratio of the costs per death between men and women, and the total cost of lost productivity as a percentage of GDP.

**Table 3:** Estimated cost per death (US$, 2019), ratio of costs per death (men to women), and total cost as % of GDP, for CVD, in South America, 2019

	Cost per death (US$, 2019)			Ratio of costs per death (M/W)	Total cost as % of GDP
	W	M	Total		
**Argentina**	20,884	28,899	26,418	1.38	0.10
**Bolivia**	26,106	33,574	30,053	1.29	0.19
**Brazil**	15,612	22,507	19,905	1.44	0.12
**Chile**	24,129	36,271	32,409	1.50	0.06
**Colombia**	13,259	21,838	19,208	1.65	0.04
**Ecuador**	32,745	49,850	43,417	1.52	0.21
**Guyana**	14,120	22,337	19,200	1.58	0.24
**Paraguay**	31,127	38,729	36,081	1.24	0.25
**Peru**	26,396	39,169	34,192	1.48	0.09
**Suriname**	17,978	26,410	23,480	1.47	0.18
**Uruguay**	28,459	38,369	35,138	1.35	0.11
**Venezuela**	NA	NA	NA	NA	NA
**South America**	**17,848**	**25,816**	**22,904**	**1.45**	**0.11**

Source: the authors.

M: men; NA: data not available; W: women.

The loss of permanent productivity in the 11 countries analyzed represented 0.11% of their combined GDP in 2019; with costs ranging from 0.04% and 0.06%, in Colombia and Chile, to 0.25% and 0.24%, in Paraguay and Guyana, respectively. The costs per death in Colombia (US$ 19,208), Guyana (US$ 19,200), and Brazil (US$ 19,905) were almost twice lower than in Paraguay (US$ 36,081) and Uruguay (US$ 35,138). For South America, the cost per death was US$ 22,904.

The estimates made by sex indicate more deaths due to CVD among men than women of working age in South America, with important variations in the men-to-women ratio, such as 1.24 in Paraguay and 1.65 in Colombia. The costs of lost productivity in PPP and nominal terms also showed significant differences. In Chile and Argentina, PPP values for men were 3.2 (US$ 239,794,767) and 3.1 (US$ 917,730,803) times higher than those for women, respectively. Bolivia showed the smallest difference in costs in PPP between sexes (US$ 131,432,052 for men and US$ 91,079,535 for women). Nominal losses in minimum wages were 3.7 and 3.2 times greater for men in Colombia and Chile (US$ 111,061,199 and US$ 131,050,476) compared to women (US$ 29,810,206 and US$ 40,661,875). Bolivia and Peru had smaller differences between men and women: 1.44 and 2.32 times the estimated values, respectively.

Alternative scenarios were constructed ( Table 4 ) based on the results in Table 2 , varying: (a) discount rate values, (b) retirement age, and (c) number of deaths; the latter using the upper and lower CI present in the GHDx.^9^

**Table 4:** Loss of productivity percentages related to the baseline scenario with variations in discount rates, retirement age, and number of deaths

Country	No discount	6% discount	Actual retirement 5 years after legal age	Absolute number of deaths (upper CI)	Absolute number of deaths (lower CI)
		(%)	(%)	(%)	(%)
**Argentina**	33	-16	48	15	-10
**Bolivia**	34	-20	41	59	-47
**Brazil**	31	-19	41	6	-6
**Chile**	28	-18	46	9	-12
**Colombia**	30	-18	50	37	-29
**Ecuador**	40	-22	35	37	-28
**Guyana**	34	-21	30	34	-28
**Paraguay**	32	-19	41	42	-32
**Peru**	43	-24	31	50	-37
**Suriname**	31	-20	32	32	-27
**Uruguay**	29	-18	45	13	-12

Source: the authors.

CI: confidence interval

Labor losses caused by early deaths were estimated using different discount rates. Losses of productivity decreased for all countries with a 6% discount. Regarding the change in retirement age, the variations were positive, pointing to an increase in costs related to permanent loss of productivity, as there was an increase in time contributed to the LF. Mortality changed using the lower and upper CI of the GHDx. When we applied the higher CI of the number of deaths, the estimated loss of productivity increased, and, with the lower one, it decreased for all of South America.

## Discussion

The results of this analysis indicate a total cost of lost productivity due to CVD, in the economies of South America, of around US$ 3.7 billion (US$ 7.8 billion in PPP), representing 0.11% of the combined GDP of these countries (ranging between 0.04% and 0.24%). Costs per death reached US$ 22,904 in 2019. With very diverse markets in terms of hours worked and wages, the comparison between GDP and productivity losses highlights the potential impact on the economic dimension and the social losses produced by these premature deaths. Regions with lower socioeconomic indices have higher mortality rates.^6 , 8^

The countries analyzed have different PPYLL and estimated loss of productivity, which can be explained by population and economic differences (GDP, LF, unemployment, and retirement age).^10 , 13 , 22^HCA has been used to calculate and interpret the costs of premature deaths in different regions and disease groups.^2 , 14 , 21^The altered scenarios suggest that local elements such as retirement age, changes in LF, and deaths associated with CVD are relevant in analyzing the results.^2 , 21^

Our proposal differed from that of Azambuja et al.,^26^who estimated the direct and indirect costs related to cases of severe CVD in Brazil, for 2004. The authors indicated an annual cost of BRL 30.8 billion, 55.2% of which would result from loss of productivity in patients over 35 years old (BRL 17,013,350,772.00). They indicated direct costs corresponding to 8% of the country's total health expenditure and 0.52% of GDP (US$ 602 billion). Considering the average exchange rate for the dollar in 2004 (BRL 3.05),^23^the estimate of US$ 5,578,147,794 for indirect costs in critically ill patients over 35 years of age is lower than that found in our study (US$ 2,233,361,946), which included a younger active population and non-severe cases. Spending as a percentage of GDP (0.12%) reflects methodological differences, but points to proportionality between the results of these studies. Databases, methodologies, uses of other costs, and discount rates may be responsible for the differences in these results.

Brazil and Argentina were the countries most affected in terms of productivity losses, highlighting the negative impact of CVD on economic development. The expression of products in PPP gives them greater homogeneity and comparability ( Table 2 ). Few studies have addressed productivity losses related to CVD in South America or even in Latin America and the Caribbean.^3 , 4 , 7 , 26^No studies were found expressing results for loss of productivity due to CVD in PPP.

Mosegui et al.^27^calculated cancer-related permanent productivity losses in South America using the same approach (HCA) and database (GBD 2019). The authors reported 192,240 deaths and 2,463,155 PPYLL. The total loss of permanent productivity was US$ 4.4 billion and US$ 9.4 billion in PPP, 0.13% of the region's GDP. The total cost per death was US$ 23,617. CVDs kill almost 4 times more people than neoplasms.^6 , 28^The costs per death did not differ greatly from those found in our study (US$ 22,904), while the ratio of this cost per death between sexes was lower (1.28) than the 1.45 estimated for CVD. Our cost/death estimates appear lower than those reported in other locations and regions.^3 , 4 , 7 , 26^Caution is necessary when comparing some findings, due to existing methodological, population, and economic differences.

Some limitations of this study should be examined. The data used to calculate the PPYLL came from GBD 2019, presented in the GBDx,^9^an appropriate alternative given the scarcity of databases and studies with adequate methodological quality in the region. The use of global health and economic data^9 , 10 , 12 , 18^as an alternative to national data, which are generally more consistent, is related to difficulties in locating them, as observed for Venezuela. According to Hofmarcher et al.,^29^multinational analyses of cost are difficult to perform, mainly due to barriers associated with identifying information.

This analysis focused on permanent loss of productivity, allowing a quantitative characterization of the burden of CVD. Temporary losses or direct health costs were not calculated.^3 , 26^Minimum wages were used instead of average earnings. Mosegui et al.^21^pointed out that, for South American countries, average and minimum wages do not express the ways in which income and salaries are organized in different groups, given the recurrent informality in labor^10,12,18^and differences between legal and actual retirement age. Although HCA is the most used method of calculating loss of productivity,^2^its critics have indicated possible biases in income patterns, where loss of productivity is undervalued in groups at a disadvantage (young people, women), with lower earnings.

For the first time, the economic burden related to CVD has been estimated in all South American countries, in an additional perspective to the analysis of the burden of the disease. The permanent loss of productivity associated with CVD shown in this study is significant, and its individual and social impact suggests an average per capita loss of US$ 22,904 in the economically active population, as well as a social loss of approximately US$ 3.7 billion and US$ 7.8 billion in PPP for South America.

## Conclusion

Socioeconomic factors influence cardiovascular health, as well as inequalities and inefficiencies in health systems. CVD impose an important economic and health burden on South American countries, leading to social and productivity losses. Our findings can be useful in the formulation and implementation of public policies and effective strategies for the prevention, treatment, and management of CVD in the analyzed countries.
